# Potent Inhibition of Macropinocytosis by Niclosamide in Cancer Cells: A Novel Mechanism for the Anticancer Efficacy for the Antihelminthic

**DOI:** 10.3390/cancers15030759

**Published:** 2023-01-26

**Authors:** Souad R. Sennoune, Gunadharini Dharmalingam Nandagopal, Sabarish Ramachandran, Marilyn Mathew, Sathish Sivaprakasam, Valeria Jaramillo-Martinez, Yangzom D. Bhutia, Vadivel Ganapathy

**Affiliations:** 1 Department of Cell Biology and Biochemistry, Texas Tech University Health Sciences Center, Lubbock, TX 79430, USA; 2 Department of Pharmacology and Neuroscience, Texas Tech University Health Sciences Center, Lubbock, TX 79430, USA

**Keywords:** niclosamide, macropinocytosis, Na^+^/H^+^ exchanger, amino acid transporter, SLC38A5, amino acid-mediated Na^+^/H^+^ exchange, intracellular acidification, peptide transport, antihelminthic

## Abstract

**Simple Summary:**

Niclosamide, a drug used to treat tapeworm infection, has been shown to have potential as an anticancer drug. The mechanism proposed for this action is the drug-induced inhibition of multiple signaling pathways. Here we uncover an additional, but novel and hitherto unknown, mechanism for the anticancer efficacy of this antihelminthic. Niclosamide forces cancer cells to nutrient starvation by blocking macropinocytosis, SLC38A5-mediated amino acid entry and H^+^-coupled nutrient transport pathways. Macropinocytosis is a process by which cells take up extracellular fluid along with the components present in the fluid, and this process is stimulated by an alkaline pH in the cytoplasm. SLC38A5 is an amino acid transporter that is stimulated by an outwardly directed H^+^ gradient. The function of niclosamide as a H^+^ channel is responsible for the newly discovered actions of this drug reported here. The drug also inhibits SLC38A5 by direct interaction with the transporter.

**Abstract:**

Niclosamide, a drug used to treat tapeworm infection, possesses anticancer effects by interfering with multiple signaling pathways. Niclosamide also causes intracellular acidification. We have recently discovered that the amino acid transporter SLC38A5, an amino acid-dependent Na^+^/H^+^ exchanger, activates macropinocytosis in cancer cells via amino acid-induced intracellular alkalinization. Therefore, we asked whether niclosamide will block basal and SLC38A5-mediated macropinocytosis via intracellular acidification. We monitored macropinocytosis in pancreatic and breast cancer cells using TMR-dextran and the function of SLC38A5 by measuring Li^+^-stimulated serine uptake. The peptide transporter activity was measured by the uptake of glycylsarcosine. Treatment of the cancer cells with niclosamide caused intracellular acidification. The drug blocked basal and serine-induced macropinocytosis with differential potency, with an EC_50_ of ~5 μM for the former and ~0.4 μM for the latter. The increased potency for amino acid-mediated macropinocytosis is due to direct inhibition of SLC38A5 by niclosamide in addition to the ability of the drug to cause intracellular acidification. The drug also inhibited the activity of the H^+^-coupled peptide transporter. We conclude that niclosamide induces nutrient starvation in cancer cells by blocking macropinocytosis, SLC38A5 and the peptide transporter. These studies uncover novel, hitherto unknown, mechanisms for the anticancer efficacy of this antihelminthic.

## 1. Introduction

Optimal nutrient supply is obligatory for cancer cells to survive and proliferate at a rate faster than normal cells. This goal is achieved by several mechanisms. Cancer cells induce the expression of not one but several nutrient transporters, which include selective transporters for glucose, amino acids and vitamins [1,2,3,4,5,6]. In addition, two rather non-selective nutrient acquisition pathways are activated in cancer cells: autophagy and macropinocytosis [7,8,9,10]. Autophagy involves degradation of relatively non-essential cellular components in autophagolysosomes, followed by the utilization of the nutrients arising from the process for survival and proliferation. Macropinocytosis, on the other hand, mediates cellular uptake of extracellular constituents such as the proteins in the interstitial fluid, followed by fusion of the pinosomes with lysosomes and then degradation of pinocytosed components, and the subsequent use of the resultant end products in metabolism. Cancer cells also reprogram metabolic pathways in such a manner that specific metabolites that are essential for cell growth and proliferation are generated at optimal concentrations from these pathways; this includes oncometabolites such as lactate, succinate, fumarate, D-2-hydroxyglutarate and homocysteine [11,12].

SLC38A5 is an amino acid transporter which has a unique transport mode [13,14,15]. It transports several neutral amino acids in a Na^+^-dependent manner, coupled to countertransport of H^+^. The directionality of amino acid transport is reversible depending on the relative magnitude of the concentration gradients for Na^+^, H^+^ and amino acid substrates. When the transporter functions in the amino acid entry mode, the transport process results in efflux of H^+^, leading to intracellular alkalinization [16]. SLC38A3, a closely related amino acid transporter, also does the same thing [17]. This feature of intracellular pH regulation by SLC38A5/SLC38A3 is one of its kind among the three dozen or so amino acid transporters known in mammalian cells. Since a slightly alkaline pH inside the cells promotes DNA synthesis and supports cell proliferation, SLC38A5 (and maybe also SLC38A3 too) is upregulated in certain cancers to support amino acid nutrition, as well as to maintain intracellular pH at a slightly alkaline side [14,15].

Macropinocytosis requires remodeling of the cytoskeletal protein actin, and this process is promoted by alkaline pH inside the cell [18]. This is the basis for the facilitation of macropinocytosis by a Na^+^/H^+^ exchanger, which alkalinizes the intracellular pH in response to the naturally occurring inward-directed Na^+^ gradient across the plasma membrane of mammalian cells, and also for the effective inhibition of macropinocytosis by the amiloride derivative ethylisopropylamiloride, a potent inhibitor of the Na^+^/H^+^ exchanger [19]. With the same logic that intracellular alkaline pH stimulates macropinocytosis, we recently investigated the potential involvement of SLC38A5, which functions as an amino acid-dependent Na^+^/H^+^ exchanger, in macropinocytosis [20]. These studies did reveal that SLC38A5 mediates amino acid-dependent macropinocytosis, thus unraveling a novel function for an amino acid transporter.

Niclosamide belongs to a class of drugs known as molluscicides/helminthicides [21,22]. This is an FDA-approved drug for the treatment of tapeworm infection. Pyrvinium is another member of this family, which is also in clinical use for a similar purpose. Numerous studies in recent years have revealed that niclosamide possesses anticancer activity and that the drug could be repurposed for cancer therapy [23,24,25,26,27]. The underlying mechanism for the anticancer efficacy of niclosamide seems to be interference with multiple signaling pathways involving Wnt, STAT3, mTORC1, NOTCH and PERK. What caught our attention in the published studies about niclosamide is the function of the drug as a H^+^ channel that facilitates the efflux of H^+^ from acidic compartments (e.g., lysosomes, endosomes) within a cell and causes intracellular acidification [28,29,30]. Based on this novel function of niclosamide, we hypothesized that the drug might inhibit macropinocytosis, both basal (dependent on Na^+^/H^+^ exchanger) and amino acid-mediated (dependent on SLC38A5). If we find that niclosamide does indeed inhibit macropinocytosis, it would offer yet another, but hitherto unknown, molecular mechanism for the anticancer efficacy of the drug. Here we tested this hypothesis using multiple breast cancer cells and pancreatic cancer cells. Our studies show that niclosamide is a potent inhibitor of both basal and amino acid-stimulated macropinocytosis. In addition, niclosamide is also a direct inhibitor of SLC38A5. Neither of these features is shared by pyrvinium, another member of the molluscicides/helminthicide family that is used, similarly to niclosamide, to treat tapeworm infection [31].

## 2. Materials and Methods

### 2.1. Materials

[2,3-^3^H]-l-Serine (specific radioactivity, >5 Ci/mmol) was purchased from Moravek, Inc. (Brea, CA, USA). [2-^3^H]-Glycine (specific radioactivity, 42.4 Ci/mmol), [3,4-^3^H]-glutamine (specific radioactivity, 50.5 Ci/mmol) and [^35^S]-methionine (Specific radioactivity 10.25 mCi/ml) were purchased from PerkinElmer Corp (Waltham, MA, USA). [^3^H]-glycyl-sarcosine (1.2 Ci/mmol) was purchased from Moravek. SNARF-1-AM and TMR-dextran were purchased from ThermoFisher (Waltham, MA, USA). Niclosamide and pyrvinium chloride were from Sigma-Aldrich (St. Louis, MO, USA).

### 2.2. Cell Lines and Culture Conditions

We used five breast cancer cell lines (all representing triple-negative breast cancer) and two pancreatic cancer cell lines. All media contained 10% fetal bovine serum. Cell cultures were tested every month for mycoplasma using a commercially available detection kit (cat. no. G238; Applied Biological Materials, Inc. Richmond, British Columbia, Canada). All cell lines used in the present study were mycoplasma-free. Three of the breast cancer cell lines and two of the pancreatic cancer cell lines were from ATCC: HCC1937 (cat. no. CRL-2336), MDA-MB231 (cat. no. CRM-HTB-26) and MDA-MB436 (cat. no. HTB-136), BxPC-3 (cat. no. CRL-1687) and HPAF-II (cat. no. CRL-1997). The SUM1315MO2 cell line was obtained from Expasy (cat. no. CVCL_5589). HCC1937 cells were cultured in RPMI-1640 medium (ATCC, cat. no. 30-2001); MDA-MB436 cells were cultured in Leibovitz’s L-15 medium (ATCC, cat. no. 30-2008), supplemented with 10 μg/mL insulin and 16 μg/mL glutathione. MDA-MB231 cells were cultured in Leibovitz’s L-15 medium. SUMO1315MO2 cells were cultured in Ham’s F12 medium containing 10 ng/mL EGF and 5 μg/mL insulin. One patient-derived xenograft (PDX) cell line, identified as TXBR-100, was provided by the TTUHSC Cancer Center. This cell line was cultured in a special medium consisting of Dulbecco’s modified Eagle’s medium and Ham’s F12 medium, in a 1 : 1 ratio, supplemented with 20 ng/mL EGF, 0.01 mg/mL insulin, 500 ng/mL hydrocortisone, and 100 ng/mL cholera toxin. The pancreatic cancer cell line BxPC-3 was cultured in RPMI-1640 medium, and the HPAF II cell line was cultured in MEM medium (ATCC, cat. no. 30-2003).

### 2.3. Intracellular pH Measurement

The methodology used to monitor intracellular pH is similar to that described in previously published reports [32,33]. Cells were grown on rectangular coverslips (9 × 22 mm) until they became confluent. These cells were then incubated with 7.5 μM SNARF-1-AM in the perifusion buffer, at an extracellular pH (pH_ex_) of 7.4 for 30 min at 37 °C, followed by a 30-min incubation in the same buffer but without the dye. This allows hydrolysis of the SNARF-1-AM ester. Two coverslips were placed back-to-back (cells facing outside) in a holder and perifused at a rate of 3 mL/min, and the fluorescence of SNARF-1 was monitored with a SLC-8100/DMX spectrofluorometer (Spectronics Instruments, Rochester, NY, USA), equipped suitably for perifusion of the coverslips at 37 °C. All measurements were made using 4-nm bandpass slits and an external rhodamine standard as the reference. Fluorescence was monitored in a continuous acquisition mode by using an excitation wavelength of 534 nm and monitoring emissions at 584 nm, 600 nm, and 644 nm. The ratio of the signals at 644 nm and 584 nm was used to monitor pH changes. The 600 nm wavelength, which is insensitive to pH, was used to evaluate the efficiency of dye loading, quenching or other artefacts. Fluorescence data were converted to ASCII format for subsequent analyses in SigmaPlot, version 13.

In situ calibration curves were generated as follows. The cells on coverslips were perifused with a high K^+^-buffer (10 mM NaCl, 146 mM KCl, 10 mM HEPES, 10 mM MES, 10 mM Bicine, 2 μM valinomycin, 6.8 μM nigericin, 5 mM glucose, pH 5.5–8.0 at 0.2 pH intervals). The K^+^ in the buffer approximated the intracellular K^+^ concentration. Nigericin sets the H^+^ gradient equal to the K^+^ gradient, with valinomycin completing the collapse of the K^+^ gradient, rendering intracellular pH (pH_in_) equal to extracellular pH (pH_ex_). The 644 nm/584 nm ratio of SNARF-1 fluorescence was determined at each pH during in situ calibrations. The 644 nm/584 nm ratios of SNARF-1 fluorescence were converted to pH using a modified Henderson–Hasselbalch equation. A nonlinear least squares analysis with SigmaPlot was used to obtain the values of p*K_a_*, *R*_min_ and *R*_max_ for SNARF-1 fluorescence. The R_min_ is the ratio observed when the dye is fully protonated, and R_max_ is the ratio of fluorescence when the dye is fully unprotonated. These in situ calibration parameters were used to calculate the pH_in_ values for each individual experiment.

### 2.4. Macropinocytosis Assay

We used the same experimental approach as described in our previous publication [20] to assay macropinocytosis. Briefly, cells were plated on coverslips, placed in wells in a 12-well plate, at a density of 1 × 10^5^ cells/well, and cultured with 5% CO_2_ at 37 °C using the culture media, recommended for the cells. The cells were allowed to reach ∼70% confluency. About 16 h prior to macropinocytosis assay, the medium was removed and fresh culture medium (without the fetal bovine serum) was added. Cells were washed thrice with a buffer consisting of 140 mM NaCl, LiCl or NMDGCl, pH 7.5, all of them containing the following as the common components: 5.4 mM KCl, 1.8 mM CaCl_2_, 0.8 mM MgSO_4_, and 5 mM glucose. Subsequently, the cells were exposed to the same respective buffers, but now containing TMR-dextran (100 μg/mL), with or without amino acids at 37 °C. Then, the cells were washed thrice with the respective buffer alone and then fixed with 4% paraformaldehyde for 5 min, washed several times with phosphate-buffered saline, and mounted using Prolong diamond with 4,6-diamidino-2-phenylindole (DAPI) as a nuclear marker. Cell images were taken using a Nikon confocal microscope, with a 60× objective and analyzed using the Nippon Ichi software. The images represent a maximum projection intensity derived from a Z-stack. The fluorescence quantification was performed by measuring the corrected total cell fluorescence (CTCF) using Image J and the following formula:

CTCF = (integrated density) − (area of cell of interest) × (mean fluorescence of background).

For groups of cells (15–20 cells/field) in an image, an outline was drawn to measure integrated density, area of the cell of interest, and mean fluorescence of the adjacent background around the cells of interest. We randomly selected five separate fields with 15–20 cells and calculated CTCF for each field; the five CTCF values were averaged.

### 2.5. Uptake Measurement

Uptake of radiolabeled amino acids was used to monitor the transport function of SLC38A5. Since SLC38A5 is a Na^+^-coupled transporter with the involvement of H^+^ movement in the opposite direction, the uptake assays were done at pH 8.5 to create an outwardly directed H^+^ gradient across the plasma membrane. As there are several Na^+^-coupled amino acid transporters, even for serine that is used as the substrate in most of the experiments in the present study, we cannot specifically monitor the function of SLC38A5 by using Na^+^-containing buffer. However, unlike other Na^+^-coupled transporters, SLC38A5 is tolerant to Li^+^ (i.e., SLC38A5 functions when Na^+^ is replaced with Li^+^) [16,34,35,36]. Therefore, we used an uptake buffer with LiCl in place of NaCl. The composition of the uptake buffer was 25 mM Tris/Hepes, pH 8.5, containing 140 mM LiCl, 5.4 mM KCl, 1.8 mM CaCl_2_, 0.8 mM MgSO_4_ and 5 mM glucose. The amino acid substrates used for SLC38A5 are also substrates for SLC7A5 (LAT1), which is a Na^+^-independent amino acid transporter; therefore, uptake via this transporter will contribute to the total uptake measured in the LiCl-containing buffer. To suppress SLC7A5-mediated uptake, the uptake buffer contained 5 mM tryptophan to compete with and block the transport of SLC38A5 substrates, which is mediated by SLC7A5; SLC38A5 does not transport tryptophan and therefore SLC38A5-mediated uptake is not affected by tryptophan. To determine the contribution of diffusion to the total uptake of amino acids, the same uptake buffer but with LiCl replaced isosmotically with *N*-methyl-D-glucamine chloride (NMDGCl) was used. The uptake was measured in two buffers [20]: (i) LiCl-buffer, pH 8.5 with 5 mM tryptophan; (ii) NMDGCl-buffer, pH 8.5 with 5 mM tryptophan. The uptake in NMDGCl-buffer was subtracted from the uptake in LiCl-buffer to determine the transport activity of SLC38A5.

### 2.6. Homology Modeling and Docking Studies

The primary sequence SLC38A5 (Q8WUX1) was obtained from the Uniport database. The sequence was input into Robetta that uses RoseTTA fold. This method overcomes the challenges inherent to X-ray crystallography and cryo-EM modeling, provides insight into protein structure in the absence of experimentally established structures, and generates accurate structural models [37]. The SLC38A5 model was created using sequences of five homologous proteins with known structures (highest sequence identity). The use of multiple templates improved the accuracy of the model. The sequence identity with the templates ranged from 13.6% to 20.8%. The five proteins are: zebra fish sodium-coupled neutral amino acid transporter SLC38A9 (Q08BA4) (PDB: 7KGV, 6C08), human potassium chloride cotransporter (Q9UHW9) (PDB: 6Y5R, 6M22), *Aquifex aeolicus VF5* leucine transporter LeuT (O67854) (PDB: 6NLE), *Geobacillus kaustophilus* cationic amino acid transporter (Q5L1G5) (PDB: 6F34, 5OQT), and *Carnobacterium* sp. *AT7* amino acid transporter asc (A8UCQ5) (PDB: 6F2W).

This theoretically deduced SLC38A5 structure was docked with niclosamide, pyrvinium and glutamine molecules using AutoDock/Vina with the USCF Chimera program [38,39]. The size of the grid was 30 × 30 × 30 (Å^3^), focusing on the apparent cavity in the structure where the templates showed ligand binding.

### 2.7. Statistics

Uptake experiments were routinely done in triplicates, and each experiment was repeated at least thrice using independent cell cultures. Macropinocytosis assays were also carried out three times, particularly in experiments where the signals were quantified. The same was true for pH measurements. Statistical analysis was performed with a two-tailed, paired Student’s *t*-test for single comparison and a *p*-value < 0.05 was considered statistically significant. Data are given as means ± S.E. For quantification of fluorescence signals in image analysis related to macropinocytosis, ANOVA followed by Dunn’s test was used to determine the significance of difference among the different groups.

## 3. Results

### 3.1. Influence of Niclosamide on Intracellular pH in TNBC Cell Lines and Pancreatic Cancer Cell Lines

**Figure 1:**
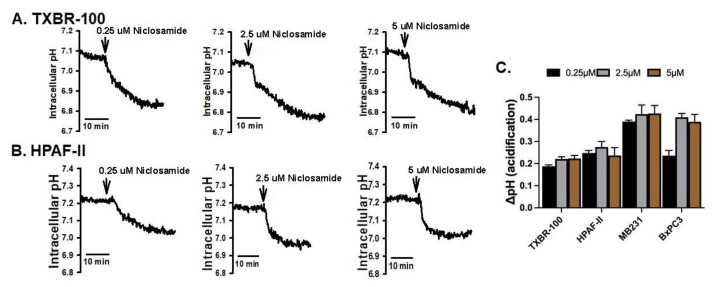
Intracellular acidification caused by niclosamide (three different concentrations) in TNBC cell line TXBR-100 (**A**) and pancreatic cancer cell line HPAF-II (**B**). The experiment was repeated in another TNBC cell line (MB231) and pancreatic cancer cell line (BxPC3). The magnitude of cellular acidification was quantified for each concentration of niclosamide; data (means ± S.E.M.) for all four cell lines are given (**C**).

Even though niclosamide has been shown to function as a H^+^ carrier and decrease intracellular pH by facilitating the release of H^+^ from various acidic organellar compartments (e.g., lysosomes, endosomes), most of these studies have focused on this effect in reference to viral entry/infection in mammalian cells [28]. Very little is known on this phenomenon in cancer cells. As such, we investigated the effects of niclosamide on intracellular pH in two TNBC cell lines (MB231, TXBR-100) and two pancreatic cancer cell lines (BxPC3 and HPAF-II). The cells were exposed to niclosamide during the measurement of intracellular pH, and the cellular pH was monitored for 30 min. Niclosamide decreased the intracellular pH in the TNBC cell line TXBR-100 (Figure 1A) and in the pancreatic cancer cell line HPAF-II (Figure 1B). The decrease in cellular pH was significant even with the lowest concentration of niclosamide examined (0.25 μM). A similar phenomenon was observed in another TNBC cell line (MB231) and pancreatic cancer cell line (BxPC3) as well. The rate of decrease was determined from the slope of the decrease in pH with time (Figure 1). The cellular pH decreased at a rate of anywhere between 0.19 ± 0.01/min and 0.41 ± 0.2/min, depending on the cell line (Figure 1C).

**Figure 2:**
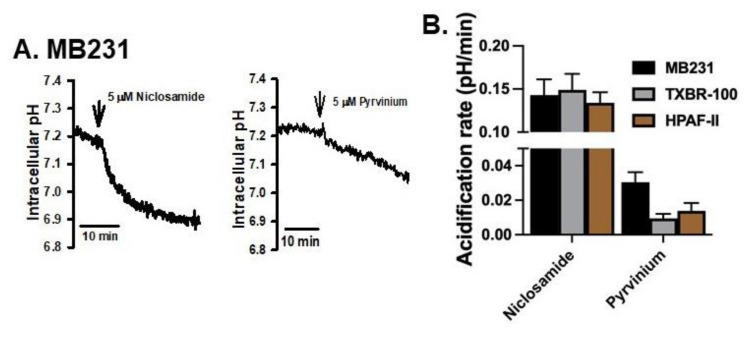
Comparison of the effects on intracellular acidification between niclosamide and pyrvinium. The time course of cellular acidification is shown in (**A**) for MB231 cells. The experiment was repeated for two additional cell lines (TXBR-100 and HPAF-II), and the magnitude of acidification for all three cell lines are shown (means ± S.E.M.) (**B**).

We then tested if pyrvinium, another pharmacologic agent used for treatment of tapeworm infection similarly to niclosamide, would elicit a similar effect in the same cancer cell lines. We performed this comparision between niclosamide and pyrvinium, both at 5 μM, in three cell lines (MB231, TXBR-100 and HPAF-II). Niclosamide decreased the cellular pH in all three cell lines, but pyrvinium showed only a minimal effect on cellular pH under identical conditions (Figure 2A,B). On average, the ability of pyrvinium to decrease intracellular pH ranged between 5–20% compared to the effect of niclosamide (Figure 2B).

### 3.2. Influence of Niclosamide on Basal Macropinocytosis in TNBC Cell Lines and Pancreatic Cancer Cell Lines

**Figure 3:**
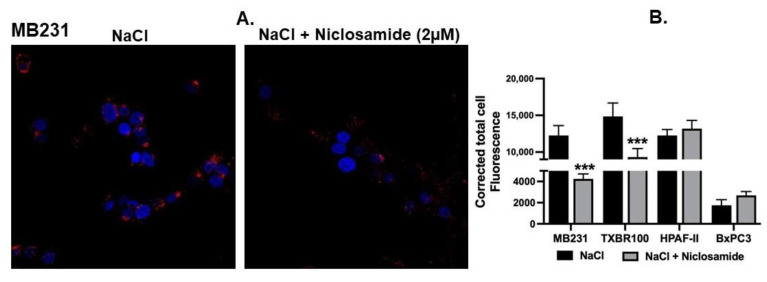
Effect of niclosamide on basal macropinocytosis. (**A**) Cellular uptake of TMR-dextran was used to monitor macropinocytosis activity in MB231 cells. The perifusion buffer contained NaCl. The assay was done in the absence or presence of niclosamide (2 μM). (**B**) The experiment was repeated in three other cell lines (TXBR-100, HPAF-II and BxPC3), and fluorescence signals were quantified as CTCF (corrected total cell fluorescence) for all four cell lines. Data (mean ± S.E.M.) are shown. ***, *p* < 0.001.

Intracellular alkalinization mediated by the activity of the plasma membrane Na^+^/H^+^ exchanger promotes macropinocytosis [18]. This represents the basal activity of macropinocytosis in cells because of the natural presence of an inwardly directed Na^+^ gradient across the plasma membrane in mammalian cells. After establishing that niclosamide does indeed decrease the intracellular pH in TNBC and pancreatic cancer cell lines, we investigated the effect of this drug on this basal activity of macropinocytosis. In the TNBC cell line MB231, niclosamide at 2 μM showed a significant effect on the macropinocytosis-mediated cellular entry of TMR-dextran (red fluorescent signals) (Figure 3A). A similar effect was seen in the other TNBC cell line (TXBR-100) though the magnitude of the effect was significantly lower in TXBR-100 cells (~35% decrease) than in MB231 cells (~65% decrease) (Figure 3B). Interestingly, this effect was not seen in pancreatic cancer cell lines (Figure 3B), even though the level of basal macropinocytosis varied between the two cell lines used in the study (HPAF-II and BxPC3) (Figure 3B).

### 3.3. Functional Evidence for Expression of SLC38A5, an Amino Acid-Dependent Na^+^/H^+^ Exchanger, in TNBC Cell Lines and Pancreatic Cancer Cell Lines

We recently discovered that the amino acid transporter SLC38A5, which mediates cellular uptake of selective neutral amino acids (glycine, serine, glutamine, asparagine, histidine, methionine) along with Na^+^, coupled to the efflux of H^+^ from the cells, is expressed at high levels in TNBC cell lines and that the transporter activates macropinocytosis [20]. In the present study, we expanded our investigation of SLC38A5 expression in TNBC to additional cell lines and also to a couple of representative pancreatic cancer cell lines. Since Li^+^ tolerance (i.e., Li^+^ is able to substitute for Na^+^ for the transport process) and increased activity of the transporter in the presence of an outwardly directed H^+^ gradient across the plasma membrane are two of the unique characteristics of SLC38A5, we monitored the uptake of serine, glycine and glutamine in the presence of Li^+^ (in place of Na^+^) and an alkaline pH in the uptake medium. Stimulation of uptake in the presence of Li^+^ compared to uptake in the absence of Li^+^ (i.e., N-methyl-D-glucamine in place of Li^+^) with an uptake buffer pH 8.5 was taken as the functional evidence for the expression of SLC38A5. These studies showed that SLC38A5 is indeed expressed in all five TNBC cell lines and two pancreatic cancer cell lines that were examined (Table 1).

### 3.4. Influence of Niclosamide on SLC38A5-Coupled Macropinocytosis in TNBC Cell Lines and Pancreatic Cancer Cell Lines

**Figure 4:**
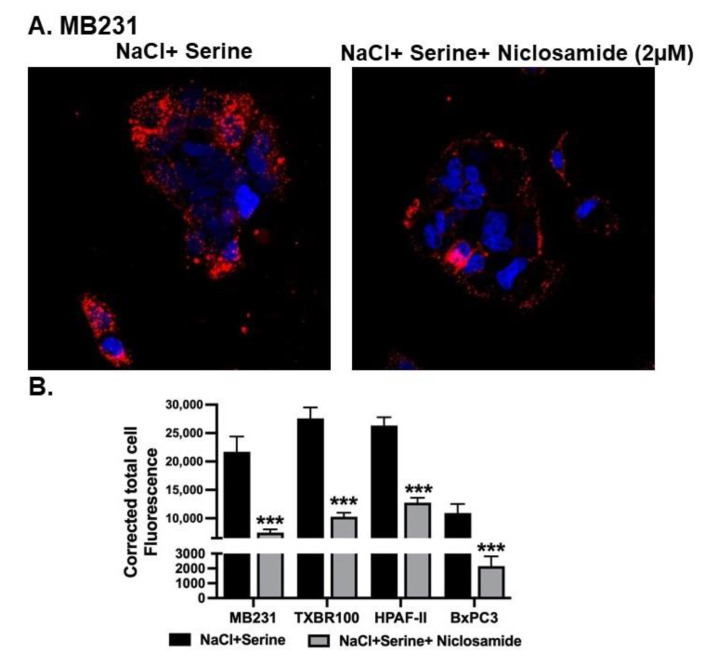
Effect of niclosamide on SLC38A5-mediated macropinocytosis. (**A**) Cellular uptake of TMR-dextran was used to monitor macropinocytosis activity in MB231 cells. The perifusion buffer contained NaCl and serine (1 mM). The assay was done in the absence or presence of niclosamide (2 μM). (**B**) The experiment was repeated in three other cell lines (TXBR-100, HPAF-II and BxPC3), and fluorescence signals were quantified as CTCF (corrected total cell fluorescence). Data are shown as mean ± S.E.M. ***, *p* < 0.001.

To determine the effect of SLC38A5 on macropinocytosis, we examined the impact of serine, a substrate for SLC38A5, on cellular uptake of TMR-dextran in a NaCl-containing medium. Addition of 1 mM serine to the extracellular medium increased the cellular uptake of TMR-dextran in all four cell lines examined; the data for MB231 cells are shown (Figure 4A). The serine-induced potentiation of macropinocytosis was evident when the uptake of TMR-dextran was compared between the data in Figure 4A and the data in Figure 3A. More importantly, niclosamide at a concentration of 2 μM showed a significant suppressive effect on the process. This blocking effect was seen not only in MB231 cells, but also in TXBR-100, HPAF-II and BxPC3 cells (Figure 4B). It is interesting to note that even though niclosamide did not have any noticeable effect on basal macropinocytosis in the pancreatic cancer cell lines HPAF-II and BxPC3 cells, it showed a marked blocking effect on serine-induced macropinocytosis.

### 3.5. Differential Efficacy of Niclosamide as a Blocker of Basal vs. SLC38A5-Coupled Macropinocytosis

**Figure 5:**
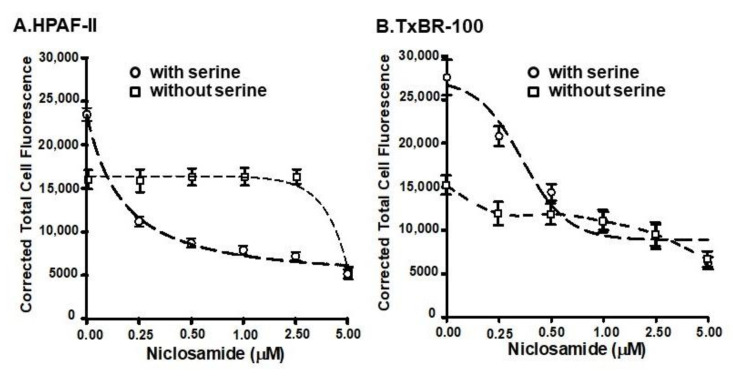
Dose-response for the inhibition of basal and SLC38A5-mediated macropinocytosis (TMR-dextran uptake) by niclosamide in HPAF-II (**A**) and TXBR-100 cells (**B**). Basal macropinocytosis was monitored in a NaCl-buffer without or with varying doses of niclosamide. The assay was done again, but using the NaCl-buffer with serine (1 mM). SLC38A5-mediated macropinocytosis was calculated by subtracting TMR-dextran uptake in NaCl-buffer from that in NaCl-serine buffer. Dose-responses for niclosamide to inhibit basal and SLC38A5-mediated macropinocytosis are shown.

Since the basal macropinocytosis and the SLC38A5-coupled macropinocytosis are mechanistically two independent processes and since both were inhibited by niclosamide, we performed a dose-response study to determine the relative efficacy of niclosamide to inhibit these two processes. This was done by quantifying fluorescence signals for TMR-dextran within the cells when cells were exposed to the fluorescent probe in a NaCl-containing buffer with and without 1 mM serine. The fluorescent signal in the presence of NaCl-containing buffer was taken as the basal macropinocytosis, and the difference in fluorescence signals between NaCl buffer and NaCl buffer plus serine was taken as the SLC38A5-coupled macropinocytosis. These experiments were done in the presence of increasing doses of niclosamide (0–5 μM) using two different cell lines (HPAF-II and TXBR-100). The data are shown in Figure 5. In both cell lines, the efficacy of niclosamide to block the basal macropinocytosis and the SLC38A5-coupled macropinocytosis was markedly different, the former being much less sensitive to blockade than the latter. The IC_50_ values for niclosamide to block the two processes were approximately 5 μM for the basal macropinocytosis and 0.4 μM for the SLC38A5-coupled macropinocytosis.

### 3.6. Direct Inhibition of SLC38A5-Mediated Transport Activity by Niclosamide, but Not by Pyrvinium

**Figure 6:**
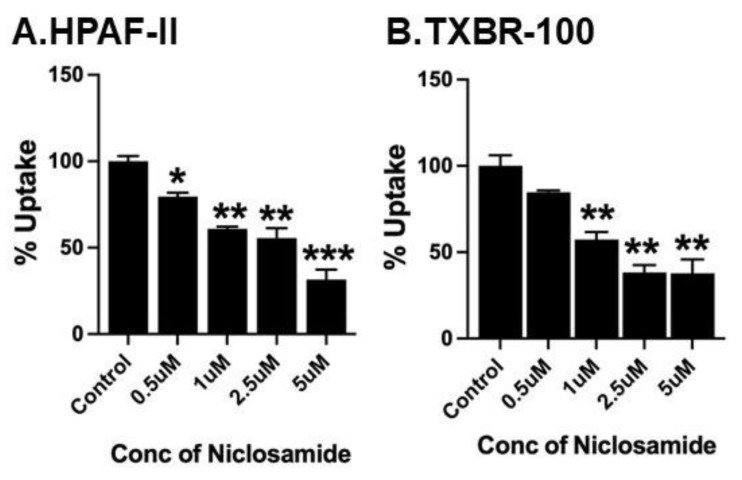
Direct effect of niclosamide on SLC38A5-mediated serine uptake. Uptake of [^3^H]-serine (0.4 μM) was measured in HPAF-II (**A**) and TXBR-100 (**B**) cells in two buffers: NMDGCl-buffer, pH 8.5 and LiCl-buffer, pH 8.5. Both buffers contained 5 mM tryptophan. Uptake was measured in the absence or presence of varying concentrations of niclosamide. The uptake in NMDGCl-buffer was subtracted from the corresponding LiCl-buffer to calculate the serine uptake that occurred via SLC38A5. Data (mean ± S.E.M.) are given as % control (i.e., uptake in the absence of niclosamide). *, *p* < 0.05; **, *p* < 0.01; ***, *p* < 0.001.

The more robust inhibition of SLC38A5-coupled macropinocytosis by niclosamide compared to basal macropinocytosis suggested that a single common pathway as the molecular target for niclosamide may not suffice to explain the differential efficacy of the inhibition of both types of macropinocytosis. This led us to examine whether niclosamide has any direct effect on the SLC38A5 transporter. For this, we monitored the effects of varying doses of niclosamide on Li^+^-stimulated serine uptake (pH of the uptake buffer, 8.5). Serine uptake that is activated by Li^+^ in the extracellular fluid was taken as the SLC38A5-specific transport activity. These experiments revealed that niclosamide is an inhibitor the transporter function; the inhibitory effect was evident in the pancreatic cancer cell line HPAF-II and the TNBC cell line TXBR-100 (Figure 6). The niclosamide inhibition was noticeable at low micromolar concentrations of the drug; the concentration needed for 50% inhibition was within the 1–2.5 μM range.

**Figure 7:**
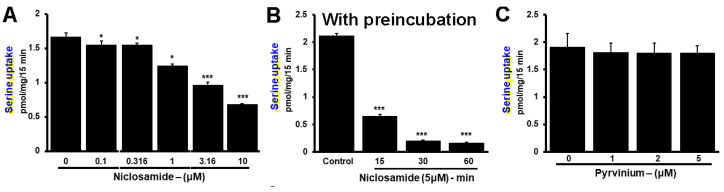
Potentiation of inhibition by niclosamide on SLC38A5 with preexposure of the cells to the drug. (**A**) Serine uptake (0.4 μM) was measured in MB231 cells with niclosamide present only during uptake measurement. SLC38A5-mediated serine uptake was calculated as described in Figure 6. (**B**) The experiment was repeated again, but this time the cells were preexposed to 5 μM niclosamide for 15, 30 or 60 min prior to initiation of uptake in the presence of 5 μM niclosamide. Thus, niclosamide was present during preexposure as well as during uptake. (**C**) Lack of inhibition of SLC38A5-mediated uptake by pyrvinium. MB231 cells were used for serine uptake in the absence or presence of varying concentrations of pyrvinium during uptake. Measurements were made in two different buffers as described in Figure 6 to allow the determination of SLC38A5-mediated uptake. *, *p* < 0.05; ***, *p* < 0.001.

Since the inhibition was observed at low micromolar concentrations of niclosamide, we asked if preexposure of the cells to niclosamide would potentiate the inhibitory effect of the drug. To address this question, we used the TNBC cell line MB231. First, we monitored the efficacy of niclosamide to inhibit Li^+^-stimulated serine uptake without preincubation (i.e., niclosamide was present only during the uptake measurement). The IC_50_ value for inhibition under these experimental conditions was within the range of 3–10 μM (Figure 7A). We then used a 5 μM concentration of niclosamide to determine the impact of preexposure. MB231 cells were exposed to niclosamide for different time periods and then Li^+^-stimulated serine uptake was measured in the presence of 5 μM niclosamide. Uptake measured in the absence of niclosamide during preincubation as well as during uptake was taken as the control. The results of the experiments showed that the efficacy of inhibition by niclosamide increased with increasing time period used for preexposure (Figure 7B). Without preincubation, the inhibition by 5 μM niclosamide was approximately 50%; the inhibition increased to approximately 90% when the cells were preexposed for 60 min to the same concentration of the drug.

Pyrvinium, a pharmacologically related drug, did not show any inhibitory effect on Li^+^-stimulated serine uptake in MB231 cells (Figure 7C). We conclude that the transport function of SLC38A5 is inhibitable by niclosamide, but not by pyrvinium.

### 3.7. Impact of Niclosamide-Induced Intracellular Acidification on the Transport Activity of SLC38A5

**Figure 8:**
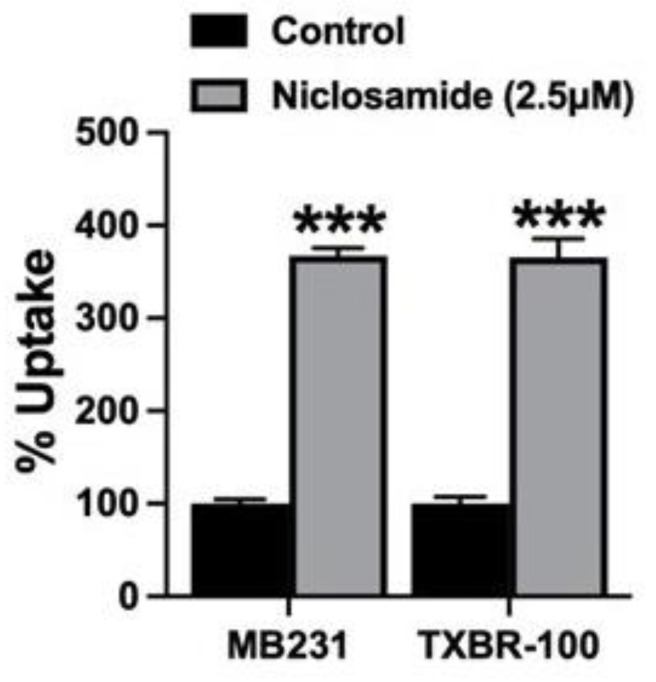
Effect of preexposure of MB231 cells to niclosamide on SLC38A5-mediated uptake. The cells were preexposed to niclosamide (2.5 μM) for 30 min in a NaCl-buffer. DMSO was used for control. Following the preexposure, cells were washed thrice with the same NaCl-buffer, but without niclosamide or DMSO. Serine uptake (0.4 μM) was then initiated in two different buffers (NMDGCl-buffer and LiCl-buffer), both at pH 8.5 and both containing 5 mM tryptophan. Niclosamide was not present during uptake. Data (means ± S.E.M) are given as percent of control (i.e., SLC38A5-specific serine uptake in cells preexposed only to DMSO). ***, *p* < 0.001.

The previously described effect of niclosamide on intracellular pH (Figure 1 and Figure 2) and the subsequently observed inhibition of SLC38A5 transport function by niclosamide (Figure 6 and Figure 7) are difficult to reconcile based on what we know about the mechanism of the transport function of SLC38A5. Since the SLC38A5-mediated transport process involves coupling between amino acid influx and H^+^ efflux, one would have expected stimulation of the transport activity of SLC38A5 by niclosamide-induced intracellular acidification. A decrease in cytoplasmic pH means increased magnitude of outwardly directed H^+^ gradient across the plasma membrane, which would provide increased driving force for the influx of amino acids (e.g., serine) via SLC38A5. Faced with this dichotomy, we realized that the experimental conditions employed in the studies described in Figure 6 and Figure 7 do not allow us to differentially monitor the direct inhibitory effect of niclosamide on the transporter and the indirect effect of niclosamide-induced cellular acidification on the transporter function. This was because niclosamide was present during uptake measurement irrespective of whether or not the cells were preexposed to the drug. To address this issue, we first preexposed MB231 cells to 2.5 μM niclosamide to cause intracellular acidification. Then, the cells were washed to remove niclosamide from the uptake buffer with subsequent uptake measurement for Li^+^-stimulated serine uptake in the absence of niclosamide. This experimental set up allowed us to monitor the influence of niclosamide-induced cellular acidification on SLC38A5-mediated serine uptake. The data from such an experiment are shown in Figure 8. Now we could detect the expected stimulatory effect of niclosamide-induced intracellular acidification on the transport activity of SLC38A5. We conclude that the efficacy of the direct inhibition of SLC38A5 transport function by niclosamide is actually underestimated because the drug has two opposing effects on the transporter; the cellular acidification stimulates the transport activity, whereas the direct interaction inhibits the transport activity. Nonetheless, the final outcome in terms of the effect of niclosamide on macropinocytosis is straightforward because the direct inhibition of SLC38A5 by the drug is also expected to cause cellular acidification because of the inhibition of H^+^ efflux from the cells. Thus, niclosamide-mediated cellular acidification is actually the combined outcome of its activity as a H^+^ carrier to release H^+^ from acidic organelles into the cytoplasm and its direct effect as an inhibitor of SLC38A5 to block the transporter-mediated H^+^ efflux from the cells.

### 3.8. Impact of Niclosamide-Induced Intracellular Acidification on the Transport Activity of the Peptide Transporter PEPT1

A couple of recently published studies reported on the expression/activity of the H^+^/peptide cotransporter SLC15A1/PEPT1 in pancreatic cancer [40,41]. This transporter mediates the influx of small peptides into cells, energized by an inwardly directed H^+^ gradient across the plasma membrane [42,43]. This transporter is Na^+^-independent, but is indirectly connected to Na^+^ via a Na^+^/H^+^ exchanger, which generates an inwardly directed H^+^ gradient across the plasma membrane in response to the normally occurring inwardly directed Na^+^ gradient [44,45]. Therefore, if niclosamide acidifies the cells, this process should have suppressive effect on the activity of the peptide transporter. To address this issue, we used HPAF-II cells, the pancreatic cancer cell line that is known to express robust activity for PEPT1 [41]. We pretreated the cells to 5 μM niclosamide for 30 min in the presence of NaCl. This was then followed by measurement of cellular uptake of the dipeptide glycylsarcosine in the absence of NaCl at pH 6.0. This uptake was compared with uptake measured in cells which were not treated with niclosamide. The data from these experiments showed that the uptake of glycylsarcosine (5 μM) was significantly lower in cells that were pretreated with niclosamide than in control cells (control, 23.6 ± 0.5 pmol/mg of protein/15 min; niclosamide preexposure, 5.0 ± 0.2 pmol/mg of protein/15 min; ~80% decrease, *p* < 0.001). These data provide additional evidence for the intracellular acidification by niclosamide and for its impact on an important nutrient transporter in cancer cells.

### 3.9. Molecular Docking Studies for Interaction of Niclosamide and Pyrvinium with SLC38A5

**Figure 9:**
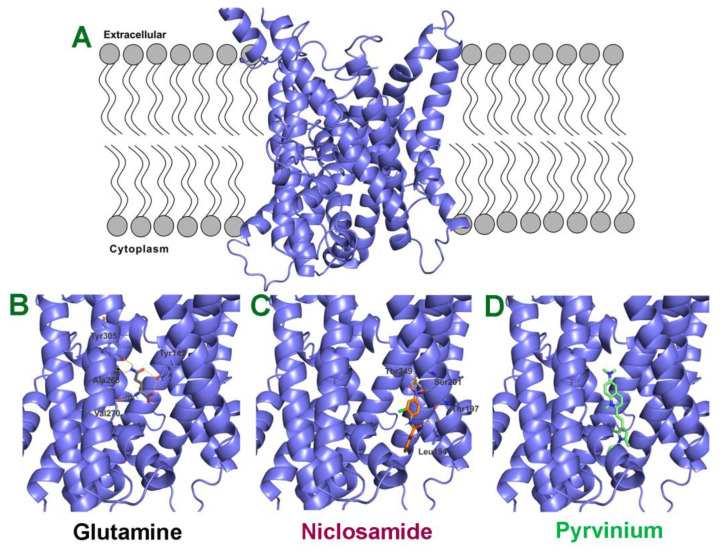
Molecular docking of glutamine, niclosamide and pyrvinium to SLC38A5. (**A**) Predicted model for SLC38A5 as it is present in the lipid bilayer. Binding of glutamine (**B**), niclosamide (**C**) and pyrvinium (**D**) to the binding pocket of SLC38A5. The ligands (glutamine, niclosamide and pyrvinium) are shown as stick models. The amino acid residues predicted to be involved in the binding of the three ligands are also shown.

The SLC38 gene family consists of 11 members, but there is no information available on the X-ray crystallographic structure or cryo-electronmicroscopic structure for any of these members. Therefore, we generated a structural model for SLC38A5 using sequences of other transporter proteins with known structures and highest sequence identity (~20%). We used five different transporters to improve the accuracy of the predicted model for SLC38A5. Molecular docking studies were then performed for niclosamide, pyrvinium and glutamine using AutoDock/Vina program. The binding energies determined from this endeavor were then used to calculate the theoretical dissociation constant for each of these three molecules. The resultant values were 3 μM for niclosamide (ΔG = −7.5 kcal/mol), 7.6 M for pyrvinium (ΔG = 1.2 kcal/mol), and 65 μM for glutamine (ΔG = −5.7 kcal/mol). Multiple amino acid residues were predicted to facilitate the binding of niclosamide (Leu194, Thr197, Ser201 and Thr349) and glutamine (Tyr142, Ala268, Val270 and Tyr305) to the transporter (Figure 9). Pyrvinium showed no interaction with the transporter, as evident from the dissociation constant in molar range and the lack of involvement of any amino acid residues in binding. These findings and the predicted values for dissociation constants corroborate to a large extent with experimental findings in the present study and in the literature. Niclosamide inhibited SLC38A5 transport activity with an IC_50_ value in the 1–2.5 μM range. Pyrvinium showed no inhibitory activity against SLC38A5. Glutamine is known to interact with SLC38A5 with a Michaelis constant of ~990 μM [46].

## 4. Discussion

In this study we have shown that the widely used antihelminthic drug niclosamide induces intracellular acidification by acting as a H^+^ carrier to facilitate the release of H^+^ from acidic organelles. However, this particular action of the drug is already known. What is new in the present study is that we have demonstrated this activity in cancer cells and, in addition, probed the relevance of the drug-induced cellular acidification to anticancer efficacy of the drug. Cancer cells produce lactic acid as the end product of aerobic glycolysis, also known as the Warburg effect, in which pyruvic acid generated in glycolysis is preferentially converted into lactic acid even in the presence of oxygen [1,47,48,49]. As a result of this cancer cell-specific metabolic shift, there is a risk of intracellular acidification in cancer cells that could prove to be fatal if not prevented. Cancer cells avoid this detrimental effect by promoting the efflux of lactic acid via the H^+^-coupled monocarboxylate transporters MCT1 (SLC16A1) and MCT4 (SLC16A3), both of which are upregulated in cancer [50,51]. There are additional mechanisms that cancer cells exploit to prevent cellular acidification [47,52]. This underscores the relevance of niclosamide-induced cellular acidification to cancer cell biology. If cancer cells regulate multiple transport systems to promote H^+^ efflux to survive in the face of cellular acidification that is caused by changes in cancer cell metabolic pathways, niclosamide-induced acidification will go against this survival strategy. This provides a novel molecular mechanism for the anticancer efficacy of this drug. Several studies have shown the anticancer effects of niclosamide in multiple experimental systems, but the underlying mechanisms reported in these studies include interference with signaling pathways involving Wnt, β-catenin and STAT3, and transcription factors such as ATF3/4, NF-κB and PERK [23,24]. There is no report in the published literature suggesting intracellular acidification as a contributor to the anticancer efficacy of niclosamide. In this regard, the present study makes an important contribution to our understanding of the pharmacological actions of this drug that are relevant to its ability to kill cancer cells.

Another important aspect related to intracellular acidification is amino acid nutrition in cancer cells. Even though aerobic glycolysis has dominated the field of cancer biology as the key aspect of metabolic reprogramming, changes in amino acid acquisition and metabolic pathways have been brought to the forefront in recent years as at least equally important to cancer cell growth and survival [1,2,3,4,5,6]. The first thing that comes to one’s mind with regard to amino acid nutrition in cancer cells is the function of amino acid transporters in the plasma membrane. Cancer cells upregulate multiple amino acid transporters as a means to increase the influx of amino acids from circulation; this includes the transporters SLC7A5 (LAT1), SLC1A5 (ASCT2), SLC6A14 (ATB^0,+^), SLC7A11 (x^-^_c_), SLC38A5 (SNAT5) and SLC43A2 [1,2,3,4,5,6]. The beneficial consequences of increased expression and activity of these transporters relate to cancer-cell survival and proliferation in multiple ways: acquisition of essential amino acids (SLC7A5 and SLC6A14), glutamine (SLC7A5, SLC1A5, SLC6A14 and SLC38A5), methionine (SLC7A5, SLC6A14 and SLC38A5), serine (SLC1A5, SLC7A5, SLC6A14, SLC38A5, and SLC43A2), and cystine (SLC7A11). These amino acids feed into several metabolic pathways, including protein synthesis, glutaminolysis, one-carbon metabolism, protein/DNA methylation, and glutathione biosynthesis. In recent years, macropinocytosis has emerged as an important mechanism for amino acid nutrition in cancer cells in which extracellular proteins such as albumin can be taken up into cells and subsequently degraded in lysosomes to provide amino acids [7,8,9,10]. This method of amino acid acquisition by cancer cells is independent of the activity of the amino acid transporters in the plasma membrane. Directly relevant to the findings in the present study is the critical role of intracellular pH in macropinocytosis. The rearrangement of actin filaments that is obligatory for the initiation and execution of micropinocytosis is facilitated by alkaline pH in the subdomains on the cytoplasmic side of the plasma membrane [18]. Therefore, the niclosamide-induced cytoplasmic acidification is a deterrent to this rearrangement of actin filaments. This is demonstrated in the present study by showing the potent efficacy of this drug in blocking macropinocytosis. As such, we conclude that niclosamide prevents amino acid acquisition by cancer cells by blocking macropinocytosis.

Among the amino acid transporters in the plasma membrane that are upregulated in cancer cells, SLC38A5 is unique because of the involvement of H^+^ efflux coupled to amino acid influx in the transport mechanism. Therefore, the entry of amino acids via this transporter results in intracellular alkalinization. As a result, the transport function of SLC38A5 potentiates macropinocytosis, which we have demonstrated in a recently published study [20]. As a corollary, one would expect the transport function of SLC38A5 to be activated by niclosamide-induced intracellular acidification. This is indeed the case if the cells are exposed to niclosamide and the function of SLC38A5 is monitored in the absence of niclosamide. However, when niclosamide is present during the measurement of SLC38A5 transport activity, the drug inhibits the transport function. This demonstrates a direct inhibitory action of the drug on SLC38A5. The inhibition is potent, with the IC_50_ value being in the low micromolar range. The direct inhibitory action of niclosamide on SLC38A5 has two important consequences with regard to amino acid nutrition in cancer cells. First, the influx of extracellular amino acids via the transporter is blocked by niclosamide. Second, inhibition of the transport activity of SLC38A5 prevents the transporter-mediated H^+^ efflux, which is expected to block SLC38A5-dependent macropinocytosis, thus preventing the amino acid acquisition in the form of extracellular proteins. This is evident from the inhibition of amino acid-induced micropinocytosis by niclosamide with much more potency than the drug’s ability to block the basal macropinocytosis.

In the present study, we have found yet another mechanism for niclosamide to interfere with amino acid nutrition in cancer cells that involves the function of the H^+^/peptide cotransporter SLC15A1/PEPT1. We have shown recently that this transporter is upregulated in pancreatic cancer and that silencing of the transporter elicits detrimental effects on cancer cell survival [41]. The tumor microenvironment contains metalloproteinases that are secreted by cancer cells as well as immune cells, and these proteinases degrade the extracellular matrix to make room for the tumor to grow. This process also generates small peptides as a result of extracellular protein degradation. The upregulation of SLC15A1 in cancer cells coupled to the naturally occurring acidic pH in the tumor microenvironment provide the optimal conditions for the cancer cells to take up these small peptides to support their amino acid nutrition. Since the function of this transporter is coupled to H^+^ influx, niclosamide-induced intracellular acidification interferes with the driving force the peptide transporter, thus decreasing its transport activity.

Taken collectively, niclosamide interferes with amino acid nutrition in cancer cells by at least three mechanisms: inhibition of macropinocytosis of extracellular proteins via intracellular acidification, direct inhibition of SLC38A5-mediated amino acid entry into cells and also inhibition of SLC38A5-dependent micropinocytosis, and interference with the driving force for SLC15A1-mediated cellular uptake of small peptides arising from degradation of extracellular proteins. With these multi-pronged actions of niclosamide, the drug is an effective inducer of amino acid starvation in cancer cells, thus providing a potent mechanism for the anticancer efficacy of the drug.

Even though we did not monitor the effects of niclosamide on intracellular pH in normal cells, it is likely that the ability of the drug to cause intracellular acidification is not unique to cancer cells; since niclosamide is a H^+^ carrier, it is expected to have a similar effect in normal cells. However, this effect may not be detrimental to the cells if the treatment is only for a short time. In fact, the influence of niclosamide on normal cells has been examined previously [53,54]. Kaushal et al. [53] have demonstrated that treatment of normal pancreatic ductal cells with 1 μM niclosamide failed to induce any detectable cell death even when the treatment was done for 12 h. Similarly, Osada et al. [54] showed that exposure of normal fibroblasts, peripheral blood monocytic cells and normal mammary epithelial cell lines with 2 μM niclosamide even for 3 days failed to induce cell death to any noticeable extent. Chronic treatment of cancer cells with niclosamide for 24–72 h even at low micromolar concentrations has been shown to cause cell death [23,24,25,26]. In the present study, the maximum duration of treatment with niclosamide was 1 h, and we did not observe any detectable cell death in any of the experiments under the conditions employed in the study. The ability of the drug to cause cytoplasmic acidification is seen almost immediately, thus representing an acute effect of the drug. This is expected because niclosamide is hydrophobic and lipid-soluble, thus having the ability to get incorporated into biological membranes and affect H^+^ flux. Niclosamide has multiple effects in mammalian cells, and many of these effects are related to blockade of signaling pathways [23,24,25,26]. The targets of niclosamide in these signaling pathways are normally upregulated in cancer cells compared to normal cells. This could be the reason for the differential effects of niclosamide on normal cells versus cancer cells in terms of cell death and cell proliferation.

## 5. Conclusions

The studies described in this manuscript provide a novel insight into the anticancer efficacy of the antihelminthic niclosamide. Most of the previously reported studies on the anticancer effects of this drug focused on intracellular signaling pathways. Here we showed that niclosamide induces cellular acidification in cancer cells and that this acidification interferes with macropinocytosis, a key nutrient acquisition pathway. Interestingly, the drug blocks basal macropinocytosis and amino acid-coupled macropinocytosis with a 10-fold difference in potency. This finding led to another important discovery: niclosamide directly interacts with the amino acid transporter SLC38A5 and inhibits the transporter function at low micromolar concentrations. Since this transporter is responsible for amino acid-coupled macropinocytosis, the direct inhibition of the transporter provides adequate explanation for the increased potency of the drug in inhibiting amino acid-coupled macropinocytosis. In addition, intracellular acidification caused by this drug also inhibits the transport activity of the peptide transporter, which has recently been shown to play a critical role in amino acid nutrition in pancreatic cancer cells. Taken collectively, niclosamide causes amino acid starvation in cancer cells by at least three mechanisms: (i) inhibition of macropinocytosis that mediates the cellular uptake of proteins present in the tumor microenvironment, (ii) inhibition of amino acid transport via SLC38A5, and (iii) inhibition of SLC15A1 that mediates the cellular uptake of small peptides generated in the tumor microenvironment by the action of metalloproteinases on extracellular proteins such as collagen. These newly discovered pharmacologic actions of niclosamide significantly expand our understanding of the anticancer activity of this drug and strengthen the argument in support of repurposing this widely used antihelminthic to treat cancer.

## Figures and Tables

**Table 1:** SLC38A5 transport activity in TNBC and pancreatic cancer cells. Values (pmol/mg/15 min) are mean ± SEM. ***-*p* < 0.001.

	NMDGCl	LiCl
**TXBR-100**		
Serine	0.56 ± 0.032	4.60 ± 0.088 ***
Glycine	0.04 ± 0.002	0.23 ± 0.007 ***
Glutamine	0.08 ± 0.004	0.62 ± 0.011 ***
Methionine	0.24 ± 0.007	0.98 ± 0.056 ***
**MB231**		
Serine	0.11 ± 0.013	0.97 ± 0.022 ***
Glycine	0.02 ± 0.001	0.06 ± 0.003 ***
Glutamine	0.03 ± 0.003	0.05 ± 0.002 ***
Methionine	0.66 ± 0.004	1.04 ± 0.010 ***
**MB436**		
Serine	0.36 ± 0.015	3.35 ± 0.094 ***
Glycine	0.09 ± 0.002	0.17 ± 0.004 ***
Glutamine	0.13 ± 0.002	0.34 ± 0.011 ***
**HCC1937**		
Serine	0.40 ± 0.005	2.07 ± 0.015 ***
Glycine	0.10 ± 0.005	0.17 ± 0.001 ***
Glutamine	0.14 ± 0.003	0.32 ± 0.007 ***
**SUM1315M02**		
Serine	0.16 ± 0.003	2.09 ± 0.074 ***
Glycine	0.03 ± 0.002	0.15 ± 0.014 ***
Glutamine	0.03 ± 0.001	0.15 ± 0.014 ***
**BxPC-3**		
Serine	0.27 ± 0.008	3.44 ± 0.094 ***
Glycine	0.07 ± 0.007	0.33 ± 0.008 ***
Glutamine	0.11 ± 0.008	0.37 ± 0.011 ***
**HPAF-II**		
Serine	0.33 ± 0.015	1.74 ± 0.075 ***

## Data Availability

The data presented in this study are available on request from the corresponding authors.
